# A review of more than 2000 cases of site-specific pelvic endometriosis rates by MRI: a guide to minimizing under/overdiagnosis non-invasively

**DOI:** 10.1186/s13244-022-01270-z

**Published:** 2022-08-08

**Authors:** Azadeh Hajati, Omid Hajati

**Affiliations:** 1 Faraparto Medical Imaging and Interventional Radiology Center, Zand BLVD, Faghihi St., Shiraz, Fars Iran; 2 Present Address: Taba Medical Imaging Center, Zand BLVD, Moadel St., Shiraz, Fars Iran; 3 Apple Inc, Cupertino, CA USA

**Keywords:** Endometriosis, Deep infiltrative endometriosis, Magnetic resonance imaging, Rate, Distribution

## Abstract

**Objectives:**

To statistically study the incidence of endometrioma and deep infiltrating endometriosis (DIE) in various anatomical sites and to illustrate the significance and potential implications for each site. Furthermore, to improve the knowledge of the community for a non-invasive diagnosis alternative to laparoscopy.

**Methods:**

This study includes 2040 patients who had been referred with clinical evidence of pelvic endometriosis. These patients had been examined and undergone transvaginal sonography by the referring gynecologists. The imaging modality used to discover and locate various anatomical locations of involvement was MRI with contrast. Two radiologists with expertise in endometriosis separately assessed the patients' MRIs and highlighted the involved areas.

**Results:**

In total, 79.1% were positive for either endometrioma or DIE. We detected both DIE and ovarian endometrioma in 78.2% of positive cases. Isolated endometrioma or DIE was present exclusively in 13.7% and 8.1% of patients, respectively. Uterosacral ligaments were detected as the most common (73.8%) site of DIE involvements and in 2.9% of cases were the sole affected location. Interestingly, very rare independent involvement of the genitourinary tract was seen in two patients.

**Conclusions:**

In this study, MRI was used to assess the likely involvement sites of endometrioma and DIE, as well as the frequency of incidences in various places and their relationships over a large dataset. Understanding the possibly involved sites, their statistics, and their co-existence can provide radiologists with a roadmap for non-invasive endometriosis diagnosis and treatment planning. These principles should hopefully assist reduce under- and overdiagnosis.

## Key points

- A single rare DIE area involvement rate could help answer unknown sources of patient complaints.
- Information of potentially impacted sites, their frequencies, and their co-existence prevent under/overdiagnosis.
- The non-endometrioma DIE rate is 10% (an important finding), which could be missed by sonography.

## Introduction

Endometriosis is a condition in which endometrial cells develop outside the uterine cavity, causing pelvic pain. The most common sites of involvement are ovaries, tubes, and pelvic peritoneum [1]. It is mainly a young women's disease (mean age 25–29 years), but it can also be seen in adolescents as well as older women but rarely in the post-menopausal state [2].

Endometriosis infiltrates the pelvic peritoneum in three ways: superficial or non-invasive (less than 5 mm depth), ovarian or cystic (endometriomas), and deep infiltrating endometriosis (DIE) [3] which is specified by more than 5 mm of invasion. The invasion can lead to fibrosis, adhesion, and ultimately the obliteration of the relevant anatomic part [4].

Laparoscopy has long been accepted as the main modality to diagnose endometriosis, although it suffers from associated risks, limitations, and costs [5]. One of the serious drawbacks of laparoscopy is the risk of missing cul-de-sac DIE which is unfortunately among the most prevalent involvement sites. MRI, on the other hand, can quickly diagnose and detect DIE in a variety of locations. Furthermore, normal anatomy which is usually distorted in DIE [6] can be better distinguished due to the multi-planar capability of this modality. MRI is extremely efficient in evaluating ovarian endometrioma and deep infiltrating endometriosis [6]; in contrast, the superficial types and loci which are difficult to be evaluated by MRI are usually non-symptomatic ones.

MRI can be used in diagnosis (presence of disease) and evaluating the extension of disease (normal versus abnormal anatomy) as well as its detailed mapping [6]. Considering its multi-focal capabilities, MRI can simultaneously evaluate different regions of the pelvic cavity for endometriosis, a multi-organ disease. As a result, it may be the ideal imaging modality for determining the operation roadmap, particularly in complicated cases involving many organs and requiring a multi-specialty procedure [7]. In the case of imprecise imaging and mapping, the right treatment and surgery could not be done [8].

Several research [9–14] have demonstrated the efficacy of MRI in evaluating patients suspected of endometriosis, although detailed statistics are either absent or the datasets for epidemiological studies are small (less than 400 cases). A collection of several studies has been reviewed in a comprehensive study [15] to evaluate the accuracy of MRI in combined 1819 cases. A statistical survey is especially important to identify rare and isolated regions of involvement that can result in a debilitating unknown source of pain for women which can be under-diagnosed.

Our work is a large-scale cross-sectional study aiming to cover rare cases of pelvic endometriosis as well as the relationship between different locations' involvement. To our knowledge, this is the first comprehensive study over a large dataset up to this point to investigate the percentage of various compartment involvement detected by MRI.

## Material and methods

This is a cross-sectional retrospective study. From May 2016 to March 2019, 2704 women were referred to our institution for endometriosis evaluation and, as a result, pre-operation mapping. Patients with endometriosis complaints, suspicious findings in other imaging modalities (transabdominal or transvaginal ultrasonography), or known cases of endometriosis were included (previous typical imaging findings in ultrasound or MRI or cases of endometriosis in surgery with signs of recurrence). The data had been routinely collected for the patients referred to the imaging center for endometriosis without any interfering protocols or processes.

Before the imaging process, patients were instructed to fast for six hours and have a semi-filled bladder and an empty bowel. Just before the picture acquisition, antimuscarinic drugs were administered (Hyoscine 20 mg intravenous). 30–40 cc intravaginal gel was utilized in the case of non-virgin patients. In addition, for individuals who had no contraindications, a paramagnetic contrast agent (Magnevist 15 cc intravascular) was employed. T2-weighted and fat-suppressed T1-weighted sequences are the best MR imaging sequences for diagnosing endometriosis. The best sequences for detecting pelvic DIE, especially for evaluating fibrotic lesions, are T2-weighted sequences without fat suppression.

Total imaging time was 17–20 min depending on the patient size. The acquired sequences include axial and sagittal T1-weighted, axial fat-suppressed T1-weighted, axial T2-weighted, sagittal, and coronal fat-suppressed T2-weighted, diffusion-weighted and ADC mapping, and axial and sagittal fat-suppressed T1-weighted sequences with contrast (Table 1).

**Table 1:** Imaging sequences that were used and their characteristics

MRI protocol	T2 TSE axial	T1 TSE axial	T1 TSE FS axial	T2 TSE sagittal	T2 FS coronal	T1 TSE FS sagittal	DWI B value 50, 400, 800 axial plain
TR/TE (ms)	3600/128	600/21	580/21	3800/130	3000/86	550/21	3800/91
Section Thickness (mm)	4.5	4.5	4.5	3.5	4.5	3.5	5
Slice Gap (mm)	1–1.5	1	1	1	0.6–1	1	1
Flip Angle (°)	150	150	150	150	150	150	–
FOV (cm)	265	270	270	260	245	245	300
Matrix	320 * 300	320 * 280	320 * 256	320 * 280	320 * 256	320 * 256	102 * 102
Bandwidth (kHz)	206	180	179	160	206	180	1440
Frequency direction	Anterior to posterior	Anterior to posterior	Anterior to posterior	Anterior to posterior	Right to left	Anterior to posterior	Anterior to posterior
NO OF Averages	3	3	2	4	2	3	4
No of images	25	25	25	23	20–24	23	25

T1 fat-suppressed axial and sagittal protocols are also used for post-contrast-enhanced MRI with the same characteristics

Contrast is often beneficial for endometriosis evaluation, particularly in individuals with a severe or extensive illness with adhesions, or in cases when the ultrasound shows compound masses and cysts. It could aid in the better visualization and tracking of pelvic organs in a deformed state caused by adhesion, as well as comprehensive mapping.

Every patient had their renal function tested before the examination, and their medical history was reviewed and evaluated for potential allergic reactions to the contrast agents. As a result, the contrast agent was administered safely in all but a tiny percentage of patients, and no adverse reactions were reported. Two radiologists with expertise in the field of endometriosis separately assessed MRI scans (blind experiment). A gynecologist who specialized in infertility and laparoscopy, as well as an expert in the field of endometriosis, referred these cases.

Reporting protocol for each patient included the complete evaluation and investigation of exactly 10 compartments. The list includes the right ovary, left ovary, ovarian fossa, uterosacral/round ligaments, retrocervical region, rectal wall, cul-de-sac, kidneys/ureters, vagina, and bladder.

For each patient, the involvement or the lack of involvement was identified concerning all ten categories and reported. Over the full length of this study, a total of 2704 cases of pelvic MRI were referred to our center for the assessment of pelvic pain or endometriosis. Among them, this study covers 2040 cases (age 12–65 years old with a mean age of 32.85) which were referred from a single endometriosis-specialized gynecology center and had been thoroughly re-evaluated.

A 1.5 Tesla MRI machine (Siemens Avanto) had been used as the scanner, and the images were reviewed by the PACS system (INFINITT VER 5.0.0.2), using cine movie and comparative techniques.

All of these patients' MRIs were examined by two radiologists by default, as requested by the referring gynecologist. Furthermore, in the event of any difference between the MRI and ultrasound, physical exams, and/or the surgery, the images were rechecked, and the reports were verified. In particular, if there was a discrepancy between the MRI imaging results and the initial diagnosis via sonography or laparoscopy, the pictures were re-evaluated, especially for the location of concern (triple checked by both radiologist and the gynecologist). Following the reevaluation and confirmation, it was discovered that these cases were only a minor portion of the dataset, and the final results were provided in their report.

## Results

Among the 2040 patients that we tested for endometriosis, 1613 (79.1%, age 13–62 years old with a mean age of 32.7) cases were positive at least in one site for endometrioma or deep infiltrative endometriosis. For further detailed analysis, the positive cases were identified and separated from the non-involved population (21%). Age distribution was also evaluated in the involved patients with the majority of them being in their 20 s and 30 s (33% and 42%, respectively). Also, 19% of patients were between 40 and 49 years old. Only 4% and 2% were under 20 and over 50 years old, respectively (Table 2).

**Table 2:** shows demographic information of the study

Demographic information	Number
Total cases	2040
Age in all cases	12–65 (mean 32.85)
Involved cases	1613 (79.06%)
Age in involved cases	13–62 (mean 32.66)
Involved (11–20)	61 (4%)
Involved (21–30)	529 (33%)
Involved (31–40)	686 (42%)
Involved (41–50)	303 (19%)
Involved (51–60)	33 (2%)
Involved (61–70)	1 (< 0.1%)

**Fig. 1:**
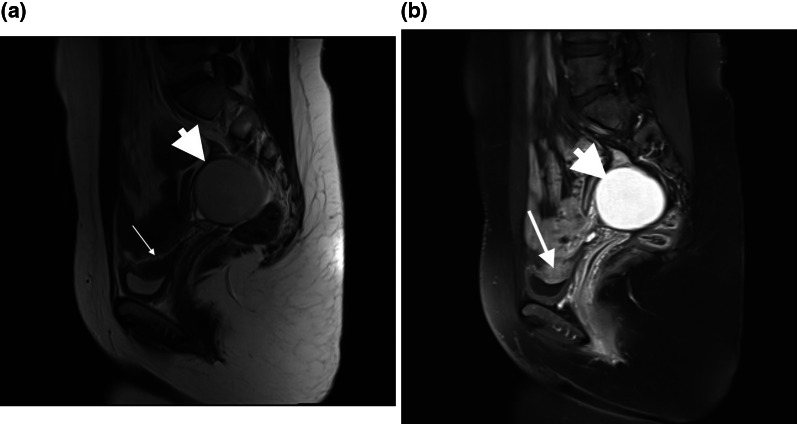
**a** T2 weighted sagittal MRI image without contrast showed a hyposignal plaque on the dome of the bladder and a typical endometrioma in the ovary (hyposignal in T2). **b** T1 weighted sagittal fat-suppressed image with contrast in the same patient showed enhancement of the plaque proved to be endometriosis in surgery, endometrioma typically hypersignal in T1

Ovarian endometrioma was detected in right, left and both ovaries in 977/1613 (60.6%), 1104/1613 (68.4%), and 599/1613 (37.1%) patients, respectively. Isolated cases of pelvic DIE in at least one site without any sign of endometrioma were confirmed in 131 (8.1%) patients. Retrocervial region, uterosacral ligaments, ovarian fossa, cul-de-sac and rectal wall involvements were diagnosed in 888 (55%), 1191 (73.8%), 419 (26%), 298 (18.5%) and 318 (19.7%) patients, respectively. Rare involvement of the vagina in 14 (0.9%) cases, as well as kidney, ureters, and bladder (Fig. 1a, b) in 11 (0.7%) patients, was diagnosed.

Endometrioma was found in at least one ovary in 1261 (78.2%) individuals with DIE (in at least one location) and 221 (13.7%) patients without DIE.

**Fig. 2:**
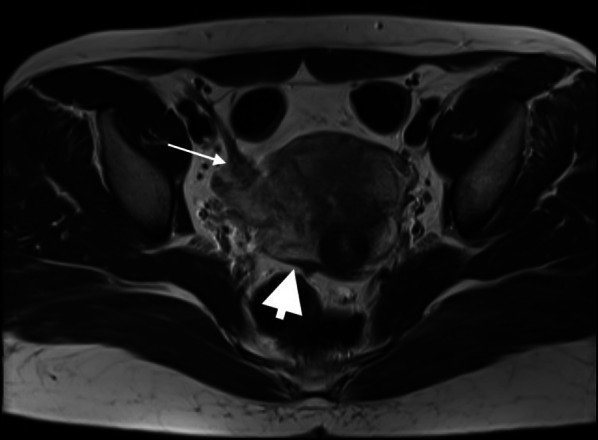
T2 weighted axial MRI image showed involvement of right uterosacral (thick arrowhead) and round ligaments (thin arrow) by endometriosis showing as nodularities, irregularities, and thickening

**Fig. 3:**
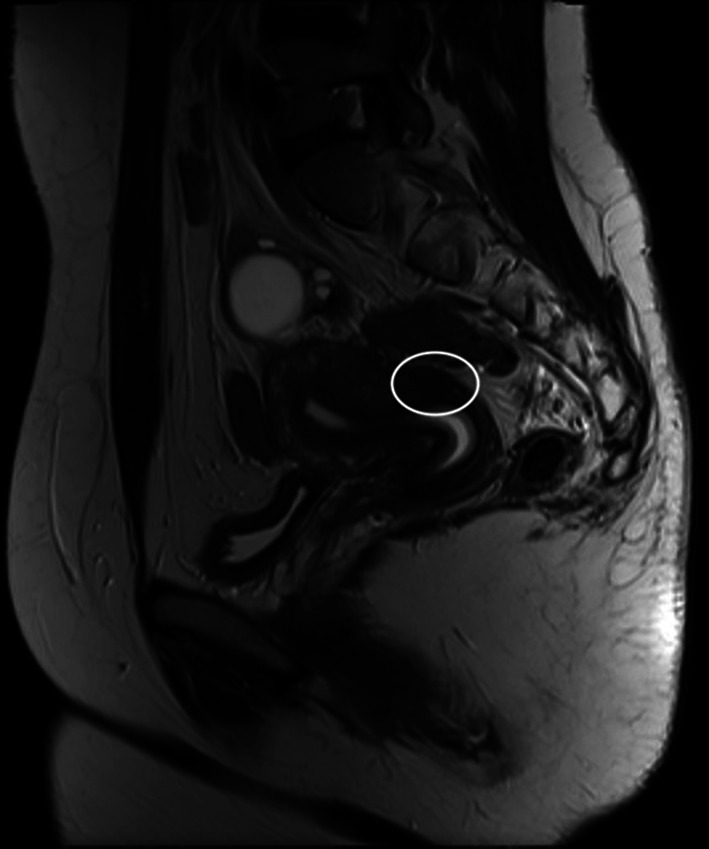
T2 weighted sagittal MRI image showed involvement of retrocervical region as a site of DIE with some adhesions in the posterior cul-de-sac

**Fig. 4:**
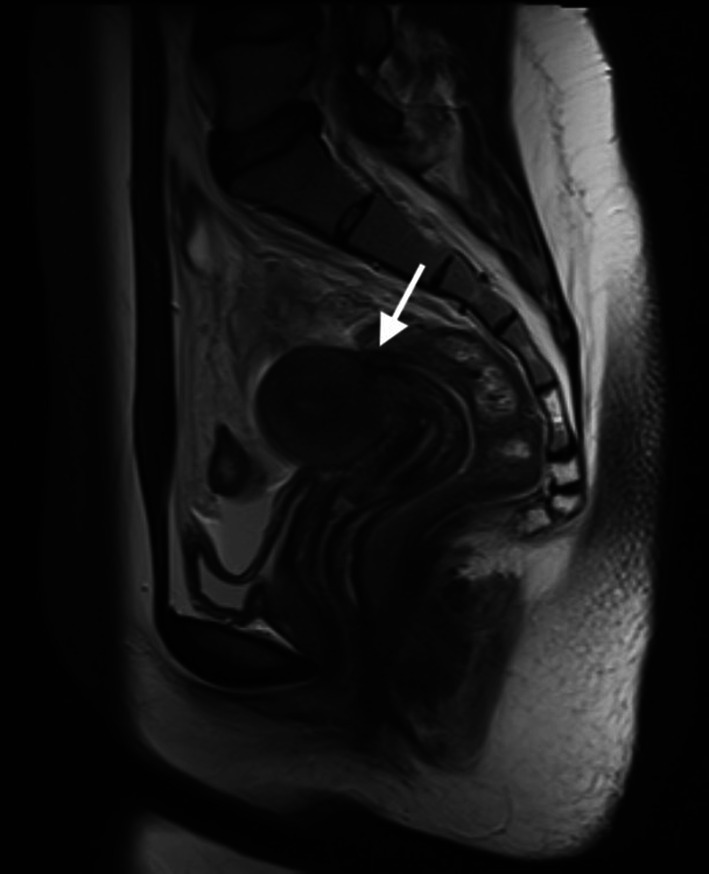
T2 weighted sagittal MRI image showed involvement of Torus-Uterinus as a single site of DIE

Isolated DIE involvements exclusively in the US ligaments, retrocervical region, and genitourinary system were found in 47 (2.9%), 15 (0.9%), and 2 (0.1%) of the cases, respectively. Ligaments were the most common site for DIE (Fig. 2), followed by the retrocervical area (Figs. 3, 4).

**Fig. 5:**
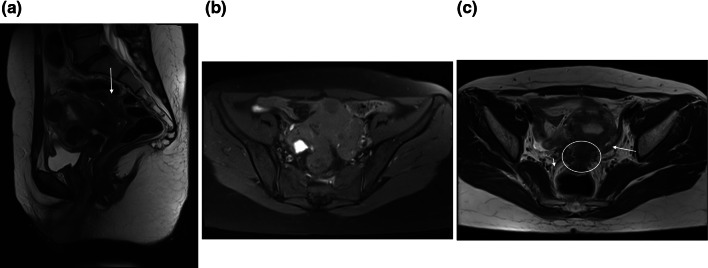
**a** T2 weighted sagittal MRI image showed large hyposignal plaque of DIE involving the rectum. **b** T1 weighted axial fat-suppressed image in the same patient shows hypersignal typical endometrioma in the right ovary. **c** T2 weighted axial MRI image showed obliteration of posterior cul-de-sac, DIE in retrocervical and rectal wall, uterosacral (small arrow) and round ligaments (large arrow) with adhesions

There was no isolated case of DIE involvement in the rectal wall (Fig. 5a–c).

As listed in Table 3, the positive cases were subcategorized based on the region of involvement in ten subcategories or body parts: right ovary, left ovary, ovarian fossa, ligaments, rectal wall, retrocervical region, vagina, cul-de-sac, kidneys/ureters, and bladder.

**Table 3:** Cases who are positive for endometriosis classified as parts that are involved

Part and type of involvement	%	Number
Endometrioma without DIE	13.7	221
Endometrioma with DIE at least in one site	78.1	1261
DIE at least in one site with no endometrioma	8.1	131
only uterosacral ligament DIE	2.9	47
only retrocervical region DIE	0.9	15
only rectal wall DIE	0	0
only kidney, ureter, or bladder or all (GU system) DIE	0.1	2
retrocervical and rectal wall DIE	17.1	276
overall DIE involvement of retrocervical region	55.0	888
overall DIE involvement of uterosacral ligament	73.8	1191
overall DIE involvement of ovarian fossa	25.9	419
overall DIE involvement of cul-de-sac	18.4	298
overall DIE involvement of rectal wall	19.7	318
overall DIE involvement of vagina	0.9	14
overall DIE involvement of kidney	0.7	11
overall DIE involvement of ureter, bladder	0.7	11
right ovary endometrioma	60.5	977
left ovary endometrioma	68.4	1104
both ovaries endometrioma	37.1	599

In addition, the patients are classified into two important groups: endometriomas groups (with and without DIE) vs DIE groups without endometrioma (Table 3).

## Discussion

Endometriosis, a common gynecological illness marked by extra-uterine endometrial cells, is the most common cause of chronic female pelvic pain [16]. Several studies have shown the accuracy of MRI for endometriosis diagnosis and DIE mapping; however, their sample sizes were insufficient, and their focus was mostly on sensitivity and specificity rather than the likelihood of involvement of specific regions. There has not been a big investigation on site-specific endometriosis involvement and their relationships till now, to our knowledge.

MRI has been found to be accurate in the diagnosis of endometriosis in numerous investigations, and various modalities have been examined. Bazot et al. evaluated the sensitivity of MRI, TVS, and rectal endoscopic sonography in 92 patients in 2009 and found that while they are equivalent in bowel loops, MRI is superior for evaluating US ligaments and vagina [9].

Luciana et al. reported on the accuracy of MRI in the bladder, retrocervical, ureters, rectosigmoid, and vagina in 2009 (92 cases) [10].

3D MRI was found to be useful in the evaluation of the bladder wall and involvement of the rectosigmoid in a study of 57 cases because it facilitates the imaging of these areas [11].

In a 2013 study (152 patients), the sensitivity and specificity of the MRI as a function of the location were evaluated and the best MRI performance was for the bladder and the worst was for the peritoneum [12].

Rectosigmoid, ovarian, and rectovaginal endometriosis can be easily diagnosed by MRI, with an excellent inter-and intra-observer agreement [13].

Despite the fact that TVS can be very specific in DIE diagnosis, it is not as sensitive as MRI [14]. For women with chronic pelvic discomfort, TVS is usually the initial step in the assessment process. DIE in the ureter, upper rectum, and rectosigmoid can be seen using both MRI and TVS. In comparison, TVS had a poorer overall sensitivity for evaluating the bladder, rectovaginal septum, vagina, uterosacral ligament, and pelvic DIE, albeit it was specific if the lesions were discovered [14]. Because of its reduced sensitivity, a negative TVS does not rule out endometriosis, and MRI should be used to map DIE before surgery [9] and in cases of complications such as malignancy.

Our research looked at how endometriosis affects different compartments and how they relate to one another. In our large database, the most common and rarest forms (2 in 2048: 0.1 percent) were identified and discussed. This could help physicians better understand the causes of pelvic or flank pain, hematuria, vaginal bleeding, and infertility in their patients.

Furthermore, the relationship between certain sites requires deeper investigation. For example, approximately one-fifth (17%) of rectal DIE is linked to retrocervical DIE; therefore, both locations should be thoroughly examined if one of them is affected. Furthermore, no single case of rectal wall DIE has been reported, so after a rectal wall DIE diagnosis, every portion of the pelvic should be re-evaluated.

Another noteworthy observation is the existence of pelvic DIE in 8.1 percent of individuals who do not have endometrioma. Because there are not any cystic lesions in these circumstances, ultrasonography may overlook them (roughly one in ten patients with endometriosis). As a result, even if there is no endometrioma, all suspicious patients should be still investigated for DIE by MRI.

**Fig. 6:**
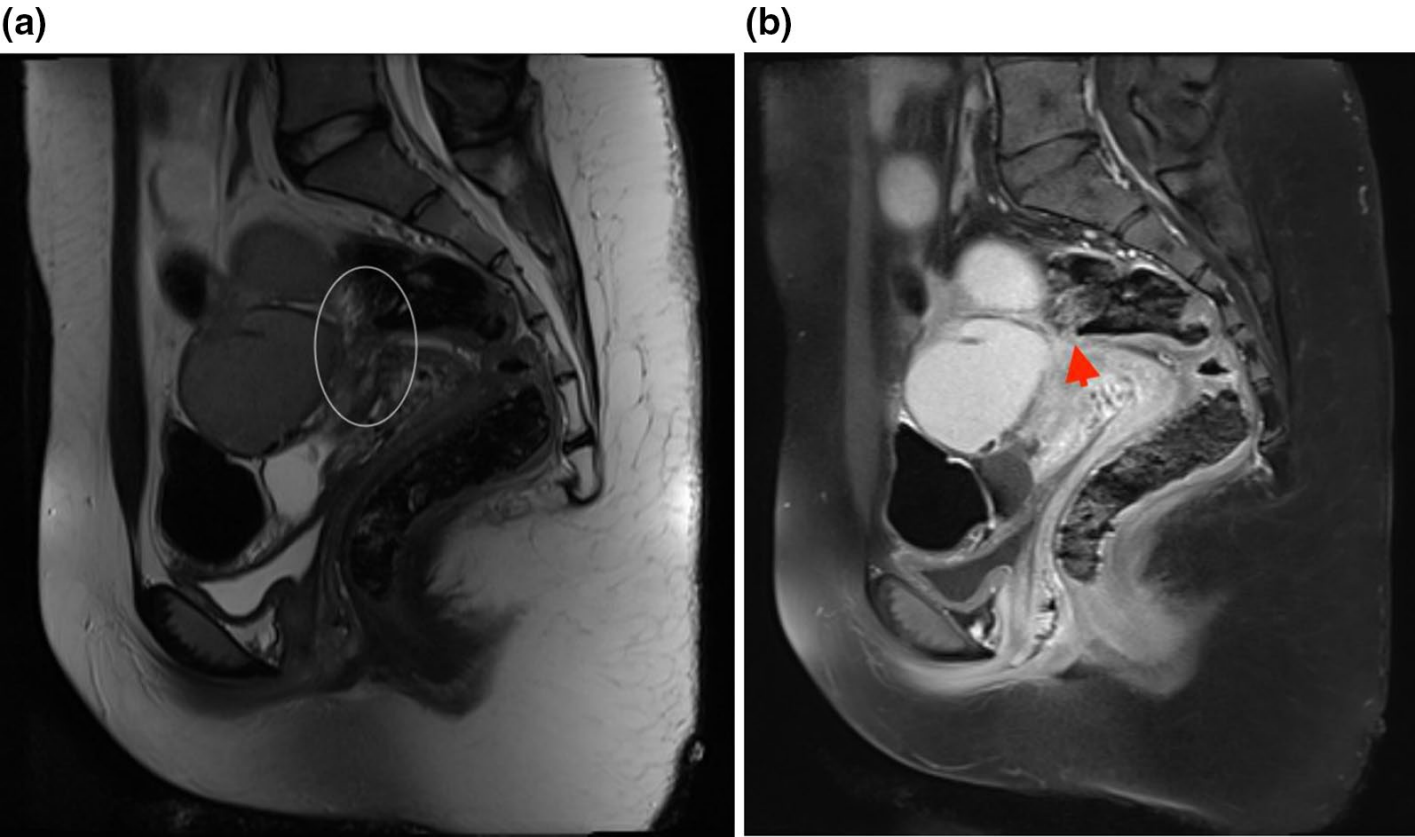
**a** T2 weighted sagittal MRI image showed involvement of posterior cul-de-sac region as a site of DIE with some adhesions with rectal wall and possibility of DIE nodule at the rectum. **b** T1 weighted sagittal fat-suppressed image with contrast in the same patient showed delineation of the rectal wall (arrowhead) with some tenting due to adhesion but no definite rectal implants

**Fig. 7:**
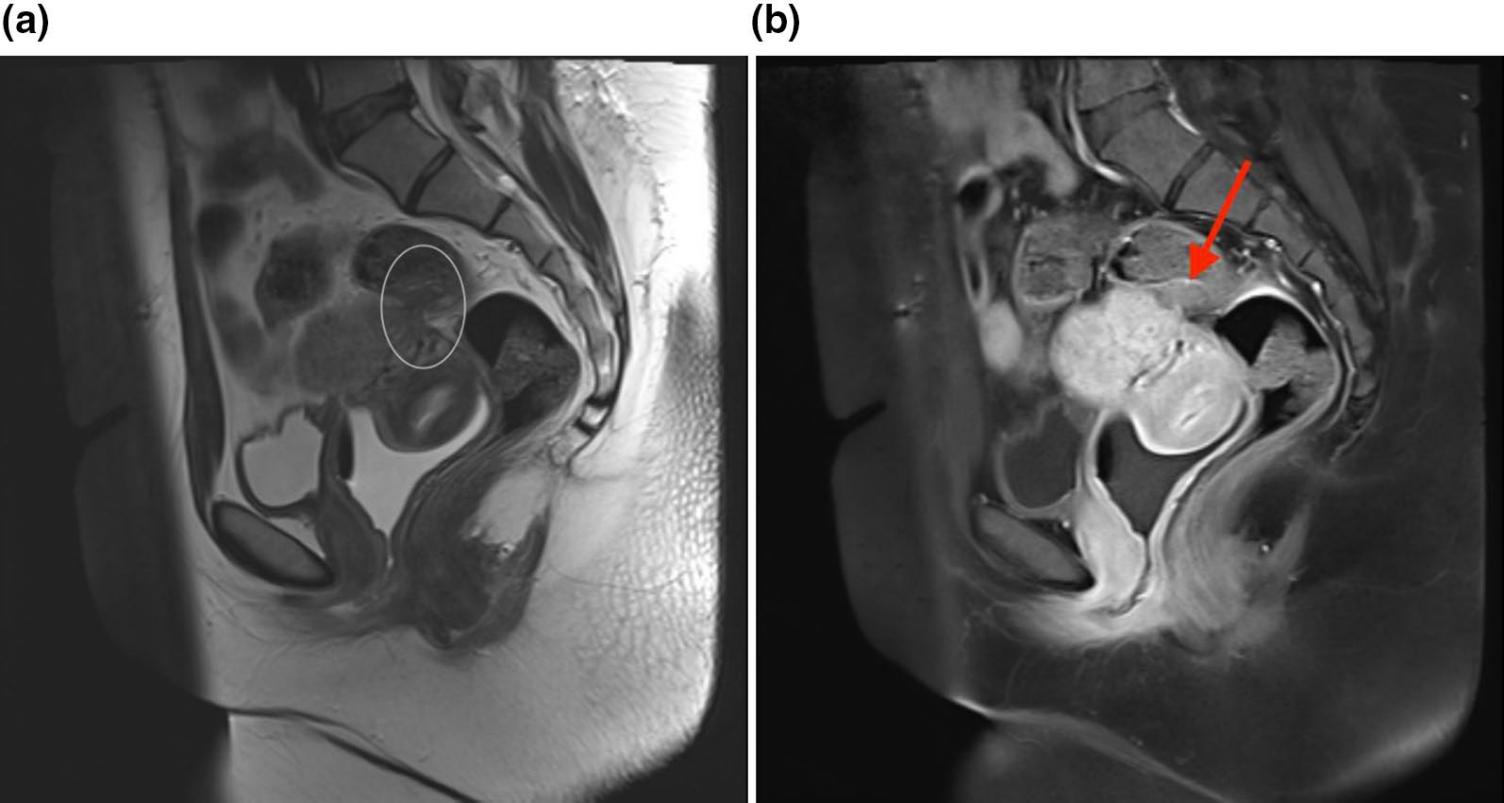
**a** T2 weighted sagittal MRI image showed involvement of retrocervical region as a site of DIE with some adhesions in the posterior cul-de-sac and rectal wall implant. **b** T1 weighted sagittal fat-suppressed image with contrast in the same patient showed rectal wall implant clearly (arrow)so size and depth can be easily measured for pre-operation planning

The contrast could have added value for endometriosis evaluation and better implant visualization (Fig. 1a, b), especially for patients with a complicated or extensive disease or cases showing compound masses in the ultrasound. It could help better delineate pelvic organs (Fig. 6a, b) in a distorted appearance occurring with adhesion and achieve a complete mapping and improve accuracy for the size of implants, (Fig. 7a, b), especially before surgery.

**Fig. 8:**
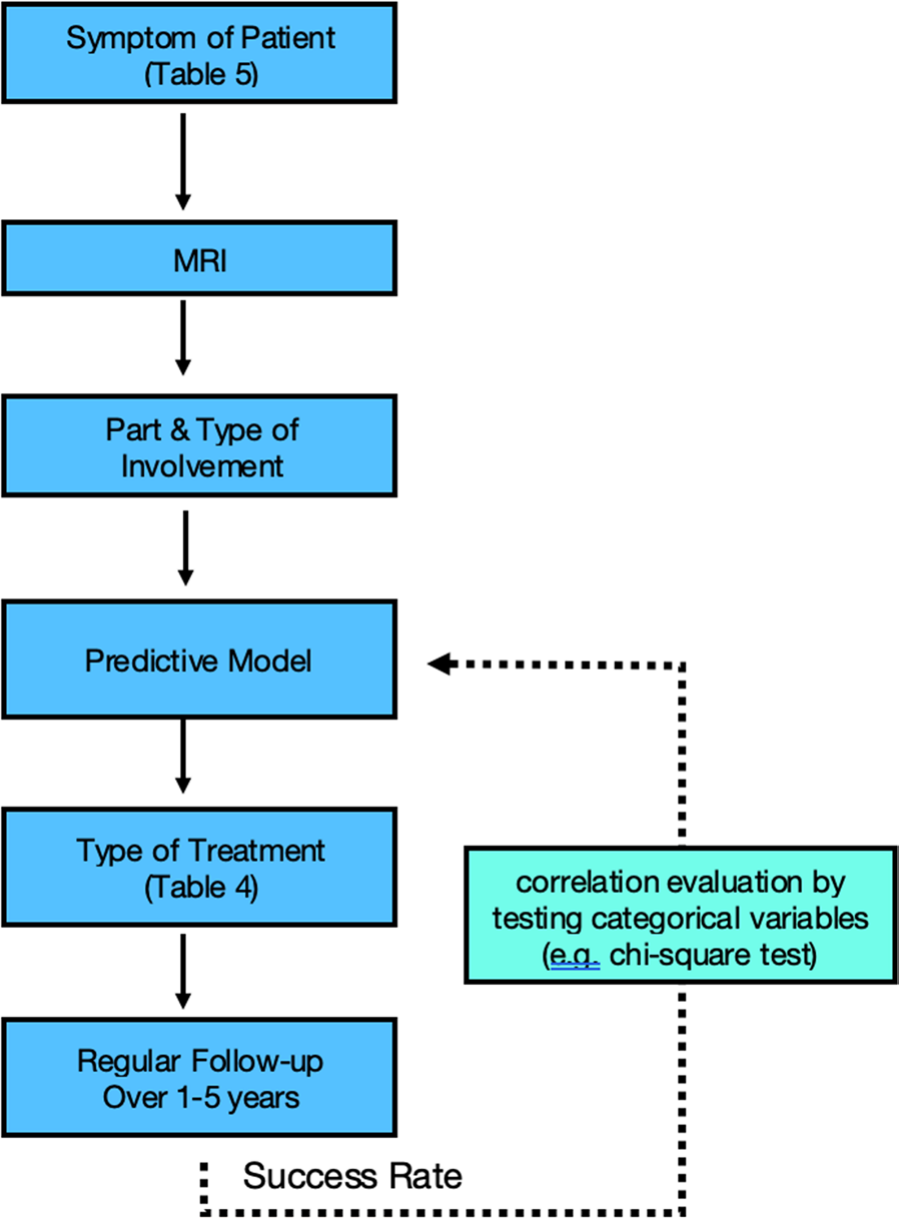
Predictive algorithm for clinical pathways based on endometriosis sites

In a recent study [17], reviewing eight most widely accepted guidelines concerning the management of endometriosis, most patients with endometriosis seek management because of pain and/or infertility. Though for the pain group, there is nearly an agreement on medical and conservative treatment as a 1st line option, there are controversies regarding infertility. Also for the 2nd and 3rd line treatments discrepancies are noted in all patients. The results of our study can be a baseline for further workups (prospective studies) to predict types of successful treatment and clinical outcomes based on the site(s) of endometriosis involvement (Fig. 8). Key parameters will be the category of treatment (Table 4) and potential symptoms of patients (Table 5). For example, only a regular follow-up without any specific treatments may be considered for asymptomatic patients with limited involvement, while a more aggressive treatment scheme has to be considered for infertile patients and extensive disease. Furthermore, the chance of complications in various endometriosis groups can be evaluated at different timescales. Hopefully, the presented statistics and insights can be used as a guideline for better management of patients with this disabling disease.

**Table 4:** Types of endometriosis treatment classification (sample)

Type of treatment
No treatment (only follow-up)
Medical treatment (e.g., hormonal tablets)
Surgery (gynecologist)
Surgery (multi-specialty)

**Table 5:** Symptoms of patients with endometriosis classification (sample)

Symptoms of patients
Asymptomatic
Pain (pelvic, dysmenorrhea, dyspareunia, dyschezia, dysuria)
Infertility
Abnormal bleeding

One of the limitations of our study was the lack of pathological confirmation in a group of patients who did not undergo surgery and instead received nonsurgical medical treatments. Nevertheless, they still underwent ultrasound (Transrectal, Transvaginal). Another limitation of our study is the evaluation of bowel loops in pelvic regions only, resulting in a smaller percentage relative to laparoscopic and pathologic studies covering the entire abdomen [9].

## Conclusion

For a large dataset based on MR imaging, the prevalence of endometrioma and DIE involvement in various pelvic compartments, as well as their statistical correlation, were assessed. Knowing the likelihood of various sections being involved as well as their co-existence is a key step in accurately diagnosing and treating endometriosis. Pretreatment DIE mapping is essential for determining the type of treatment and ensuring thorough removal, especially if a multi-specialty operation is required. Furthermore, it aids in diagnosis and prevents under- or over-diagnosis.

## Data Availability

All data and materials are accessible.

## Abbreviations

ADC: Apparent diffusion coefficient
DIE: Deep infiltrating endometriosis
PACS: Picture archiving and communication system
TVS: Transvaginal sonography
US ligament: Uterosacral ligament

## References

[1] Endometriosis, Symptom, and causes; Mayo Clinic Website (2019). https://www.mayoclinic.org/diseases-conditions/endometriosis/symptoms-causes

[2] Dmowski WP, Lesniewicz R, Rana N, Pepping P, Noursalehi M (1997). Changing trends in the diagnosis of endometriosis: a comparative study of women with pelvic endometriosis presenting with chronic pelvic pain or infertility. Fertil Steril.

[3] Samreen JN, Bookwalter CA, Burnett TL, Feldman M, Sheedy SP, Menias C, VanBuren WM, Kabashi A (2019). MRI of endometriosis: a comprehensive review. Appl Radiol.

[4] Thalluri AL, Knox S, Nguyen T (2017). MRI findings in deep infiltrating endometriosis: a pictorial essay. J Med Imaging Radiat Oncol.

[5] Taylor HS (2019) Diagnosing endometriosis: is laparoscopy the gold standard? www.mdedge.com/obgyn; http://mdedge-files-live.s3.us-east-2.amazonaws.com/files/s3fs-public/obgm_0419_endo_final_approved.pdf

[6] Waesberghe JHP, Hazewinkel M, Busard M (2011) Endometriosis—MRI detection; Radiology Assistant. https://radiologyassistant.nl/abdomen/endometriosis-mri-detection/

[7] Foti PV, Farina R, Palmucci S, Vizzini IA, Libertini N, Coronella M, Spadola S, Caltabiano R, Iraci M, Basile A, Milone P (2018). Endometriosis: clinical features, MR imaging findings and pathologic correlation. Insights Imaging.

[8] Coutinho A, Bittencourt LK, Pires CE, Junqueira F, de Oliveira Lima CM, Coutinho E, Domingues MA, Domingues RC, Marchiori E (2011). MR imaging in deep pelvic endometriosis: a pictorial essay. Radiographics.

[9] Bazot M, Lafont C, Rouzier R, Roseau G, Thomassin-Naggara I, Daraï E (2009). Diagnostic accuracy of physical examination, transvaginal sonography, rectal endoscopic sonography, and magnetic resonance imaging to diagnose deep infiltrating endometriosis. Fertil Steril.

[10] Chamié LP, Blasbalg R, Gonçalves MO, Carvalho FM, Abrão MS, de Oliveira IS (2009). Accuracy of magnetic resonance imaging for diagnosis and preoperative assessment of deeply infiltrating endometriosis. Int J Gynecol Obstet.

[11] Giusti S, Forasassi F, Bastiani L, Cela V, Pluchino N, Ferrari V, Fruzzetti E, Caramella D, Bartolozzi C (2012). Anatomical localization of deep infiltrating endometriosis: 3D MRI reconstructions. Abdom Imaging.

[12] Krüger K, Behrendt K, Niedobitek-Kreuter G, Koltermann K, Ebert AD (2013). Location-dependent value of pelvic MRI in the preoperative diagnosis of endometriosis. Eur J Obst Gynecol Reprod Biol.

[13] Saba L, Guerriero S, Sulcis R, Ajossa S, Melis G, Mallarini G (2010). Agreement and reproducibility in identification of endometriosis using magnetic resonance imaging. Acta Radiol.

[14] Indrielle-Kelly T, Frühauf F, Fanta M, Burgetova A, Lavu D, Dundr P, Cibula D, Fischerova D (2020). Diagnostic accuracy of ultrasound and MRI in the mapping of deep pelvic endometriosis using the International Deep Endometriosis Analysis (IDEA) consensus. BioMed Res Int.

[15] Medeiros LR, Rosa MI, Silva BR, Reis ME, Simon CS, Dondossola ER, da Cunha Filho JS (2015). Accuracy of magnetic resonance in deeply infiltrating endometriosis: a systematic review and meta-analysis. Arch Gynecol Obstet.

[16] Bourgioti C, Preza O, Panourgias E, Chatoupis K, Antoniou A, Nikolaidou ME, Moulopoulos LA (2017). MR imaging of endometriosis: spectrum of disease. Diagn Interv Imaging.

[17] Kalaitzopoulos DR, Samartzis N, Kolovos GN, Mareti E, Samartzis EP, Eberhard M, Dinas K, Daniilidis A (2021). Treatment of endometriosis: a review with comparison of 8 guidelines. BMC Womens Health.

